# Old Masters of Medicine

**Published:** 1908-01-25

**Authors:** 


### THE EVOLUTION OF MEDICINE.

### OLD MASTERS OF MEDICINE.

### II. CLAUDIUS GALENUS AND HIS SYSTEM.

THE Alexandrian school was strictly Aristotelian in its leanings. Herophilus, the Darwin of his day, had, it is true, made observation and deduction the guiding lines in his teaching. He had taught that the arteries conveyed both blood and pneuma; and his successor, Erasistratus, had gone even further, declaring the brain, and not the umbilicus, to be the centre of the nervous system, differentiating between motor and sensory nerves, demonstrating the finer arterioles, and almost forestalling Harvey but for the fact that he still inclined to the old belief—a belief his predecessor had opposed—that the arteries conveyed only the pneuma and had no connection with the veins.

Galen must have amassed a large fund of professional knowledge at Alexandria. His book on Erasistratus has been lost, but his works on the systole of the heart, on the pulse, and on fevers embody the orthodox Alexandrian principles. Here, too, he must have obtained his close insight into the systems of Egyptian medicine, which induced him to travel farther afield, to visit Syria, Egypt itself, and Arabia. As a separate and independent system, Arabian medicine was not yet evolved, but the clinical experience he gained from his Arabian teachers was valuable in the extreme. He observed their methods closely, and pays a high compliment to their skill, in striking contrast to the "jugglery, irrational empiricism, and clotted nonsense" which he found in the sacred book "Embre," the work of the Egyptian physician Imhotep.

In his twenty-eighth year Galen returned home, and lost no time in profiting by his clinical experience. Elected to the chair of physics in the school attached to the Temple of Asclepius in Pergamus, he began to lecture on medical subjects, working assiduously in his spare time at physiology. Then suddenly, and without assigning any reason for the step, he resigned his appointment, after having occupied the chair for six years, and went to Italy. A great deal of speculation exists as to the cause that induced him to resign. His enemies assert, with some show of reason, that it was his innate cowardice that prompted the step. He was admittedly a person who possessed little physical courage, and though no one can deny that he had a large share of moral vigour, there are incidents in his career that reflect little credit on him as a man. But more probably his resignation was due to his sincere desire to travel once more. The fame of the Italian schools had already reached his ears, and had probably aroused in him an eagerness to investigate the new doctrines for himself.

He fixed upon Rome as his place of residence, and began to practise as a physician. His striking personality, handsome, grave, and dignified in manner, his ability as a disputant, his charm as a speaker, and, above all, his singular success as a practical physician, rapidly won him a large and influential practice. He counted among his patients members of the aristocracy, consuls and senators, but made a point of attending his poor patients as assiduously as his better-class clientele. He prided himself upon his prognostic skill, claiming never to have made a false prognosis, and his reputation extended throughout Italy. As his practice became larger and more lucrative, and his influence as a teacher extended, he began to lecture on anatomy and physiology, performing experiments on animals and demonstrating the functions of the body and the structure of the various organs. In this manner he rapidly gained fame as a teacher. With his Latin colleagues he appears to have been on terms of armed neutrality. His disagreement with the Erasistratians, the Empirics, and the Methodists he never scrupled to express in strong terms, highly tinged with contempt and pungent satire. These sects—and it must be remembered that the majority of practitioners in Italy belonged to one or other—he declared were fools, buffoons, irrational lunatics, numskulls, cheats, murderers, criminals of the blackest dye. No word was too strong for them, no contempt too scathing. Under those circumstances it is not surprising that his fellow-practitioners should have repaid him in his own coin. According to them, he was unscrupulous, avaricious, and grasping, a conglomeration of exaggerated vanity, pomposity, and ineptitude, socially a rake, professionally a quack and an ignoramus. Such professional amenities make amusing reading for the student nowadays, and throw a vivid light on the state of the profession in those days. When Galen accused the Roman physicians of poisoning their successful foreign competitors, and when his colleagues retorted by vilifying him, it is difficult for the modern reader to judge between them. No one reading Galen's works, however, can fail to be struck by the fact that he was in many ways a vain man. His inflated style, his continual allusions to personal triumphs in diagnosis, usually obtained at the expense of his colleagues, and the overweening egotism which he displays are, to say the least, undignified in one who was styled "The Master."

We know little about his later years. It appears, however, that he left Rome suddenly—as abruptly as he quitted Pergamus—and returned to his native place. Here he practised with great success for several years, and then journeyed to Rome once more. He accompanied Marcus Aurelius on one of the latter's campaigns, and was, at his own request, appointed body surgeon to the young Commodus, heir to the co-Emperor Verus, drawing a princely salary as such. In his old age he became less aggressive, even friendly disposed, towards his colleagues who differed from him, and, in fact, became quite popular in the profession. He still lectured, dissected, and practised, dying in 201, at the height of his fame as a teacher and a physician. *(To be continued.)*

